# The impact of methamphetamine use on medications for opioid use disorder (MOUD) treatment retention: a scoping review

**DOI:** 10.1186/s13722-023-00402-0

**Published:** 2023-08-16

**Authors:** Cayley Russell, Justine Law, Sameer Imtiaz, Jürgen Rehm, Bernard Le Foll, Farihah Ali

**Affiliations:** 1 Centre for Addiction and Mental Health (CAMH) & Ontario Node, Institute for Mental Health Policy Research, Canadian Research Initiative in Substance Misuse (CRISM), 33 Ursula Franklin St., Toronto, ON M5S 2S1 Canada; 2 Dalla Lana School of Public Health, University of Toronto, Toronto, ON M5S 1A1 Canada; 3 Department of Psychiatry, University of Toronto, Toronto, ON M5S 1A1 Canada; 4 Institute of Medical Science (IMS), University of Toronto, Toronto, ON M5S 1A1 Canada; 5 Institut Für Klinische Psychologie Und Psychotherapie, Technische Universität Dresden, Chemnitzer Str. 46, 01187 Dresden, Germany; 6 Department of International Health Projects, Institute for Leadership and Health Management, I.M. Sechenov First Moscow State Medical University, Bol’shaya, Pirogovskaya Ulitsa, 19c1, Moscow, Russia 119146; 7 Center for Interdisciplinary Addiction Research (ZIS), Department of Psychiatry and Psychotherapy, University Medical Center Hamburg-Eppendorf (UKE), Martinistraße 52, 20246 Hamburg, Germany; 8 Department of Pharmacology and Toxicology & Department of Family and Community Medicine, Faculty of Medicine, University of Toronto, Toronto, ON M5S 1A1 Canada; 9 Translational Addiction Research Lab, Campbell Family Mental Health Research Institute, Centre for Addiction and Mental Health (CAMH), Toronto, M5S 2S1 Canada; 10 Waypoint Research Institute, Waypoint Center for Mental Health Care, Penetanguishene, ON L9M 1G3 Canada

**Keywords:** North America, Methamphetamine, Medications for opioid use disorder, Opioid agonist treatment, Opioid use disorder, Treatment retention

## Abstract

**Background:**

An emerging public health threat of methamphetamine/opioid co-use is occurring in North America, including increases in overdoses related to concomitant methamphetamine/opioid use. This presents a potential risk to established treatments for opioid use disorder (i.e., medications for opioid use disorder [MOUD]). To date, few studies have examined the impact of methamphetamine use on MOUD-related outcomes, and no studies have synthesized data on MOUD retention.

**Methods:**

A scoping review was undertaken to examine the impact of methamphetamine use on MOUD retention. All original published research articles were searched in Embase, MEDLINE, PsychINFO, CINAHL, Scopus, Cochrane Central Register of Controlled Trials, Cochrane Database of Systematic Reviews and Cochrane Protocols, and Google scholar databases. Data were extracted into a standardized data extraction chart. Findings were presented narratively.

**Results:**

All eight included studies demonstrated an increased likelihood of treatment discontinuation or dropout among patients enrolled in MOUD who used methamphetamine. The frequency of methamphetamine use was also associated with MOUD dropout, in that those who used methamphetamine more often were more likely to discontinue MOUD. The definitions and measurements of MOUD retention varied considerably, as did the magnitude of effect size.

**Conclusions:**

Results indicate that methamphetamine use has an undesirable impact on MOUD retention and results in an increased risk of treatment discontinuation or dropout. Strategies to identify concurrent methamphetamine use among individuals engaging in MOUD and educate them on the increased risk for dropout should be undertaken. Further research is needed to understand how MOUD retention among patients with concomitant opioid and methamphetamine use can be improved.

**Supplementary Information:**

The online version contains supplementary material available at 10.1186/s13722-023-00402-0.

## Background

Opioid use has been on the rise over the past decades in North America and has resulted in an unprecedented increase in opioid-related harms, including poisoning deaths, which have reached a new record high in both the United States (US) and Canada in recent years, largely due to increases in synthetic and illicitly manufactured opioids, such as fentanyl and related analogues [1, 2]. For instance, over 100,000 individuals died from drug poisoning in the US in 2021, where synthetic opioids (including fentanyl) accounted for nearly two-thirds (64%) of these deaths [3]. Similarly, in Canada, fentanyl accounted for 86% of the 26,690 opioid poisoning deaths that occurred within the first nine months of 2021 [1]. Poisoning deaths involving psychostimulants such as methamphetamine and cocaine have also increased in the US and Canada, both with and without opioid co-involvement [4]. For instance, in the US, opioids were indicated in over half (54%) of stimulant poisoning deaths in 2019, which is an increase of more than double that of 2010 [4]. In addition, more than half (58%) of opioid poisoning deaths in Canada between January to September 2021 also involved a stimulant, 53% of which also involved methamphetamine specifically. [1]

Additional data point to an increase in methamphetamine use, methamphetamine and opioid co-use, as well as methamphetamine and opioid concomitant treatment episodes in the US and Canada [5–9]. For instance, past-year methamphetamine use increased from 22.5% to 37.4% among US-based individuals with past-year heroin use (2015–2018) [10–12]. Notably, among those seeking treatment for opioid use disorder (OUD) in the US, methamphetamine use increased from 18.8% to 34.2% between 2011 and 2017. [13] Similarly, heroin use also increased from 5.3% to 23.6% among those seeking treatment for methamphetamines (2008–2017). [14] While comparable national Canadian data are limited, extant province-specific information indicates that the prevalence of methamphetamine use has increased significantly among individuals seeking treatment or visiting harm reduction services in select jurisdictions [7]. For instance, a recent study demonstrated an increasing trend in amphetamine-related emergency department and inpatient visits in the city of Toronto, Ontario, including high rates of co-occurring psychiatric disorders and opioid use [15]. Moreover, among clients of harm reduction services in the province of British Columbia (BC), crystal methamphetamine was the most frequently reported substance used in 2018 (59.7%) and 2019 (71.7%), and there was a threefold increased odds of crystal methamphetamine use among individuals who use opioids [16]. These data underscore the evolving nature of the opioid epidemic, which poses an increasingly complex public health issue.

Reasons and motivations for the rise in methamphetamine and opioid co-use have been examined qualitatively and have pointed to a number of explanations. These include to enhance one’s ‘high’ or to prolong the intoxication effect of opioids and corresponding time to experiencing withdrawal, to balance or counterbalance the effects produced by each drug, to replace or substitute opioids due to a decrease in opioid availability and ease of access to methamphetamines, to reduce chronic pain or emotional distress, and to self-treat symptoms of opioid withdrawal. [13, 17–20]

In North America, guidelines recommend that OUD is primarily treated by way of medications for opioid use disorder (MOUD), which typically include methadone and/or buprenorphine/naloxone, although alternative pharmacotherapies also exist and are used in different jurisdictions. For instance, extended-release buprenorphine formulations, slow-release oral morphine, and diacetylmorphine are all used—albeit to a lesser degree—in Canada, while opioid antagonists such as naltrexone have been approved for use in the US [21]. MOUD is considered the gold standard treatment for OUD and has been proven effective at reducing illicit substance use, drug-related crime, morbidity (e.g., HIV, HCV), and mortality [21, 22]. However, MOUD treatment engagement and retention remain typically low, and it is estimated that only a small percentage of those with OUD initiate MOUD, and less than half of those who do enter treatment remain engaged in it for more than 6 months. [22–25] For example, a recent retrospective study that examined all individuals in BC who received at least one MOUD dispensation between 2008 and 2018 found that less than 60% completed induction, and only half of those reached the minimum effective dosage [26]. Numerous barriers to MOUD have been identified in the literature [27, 28], and clients typically fall into cyclical patterns of MOUD engagement, disengagement, and re-engagement [26]. However, observational studies have shown the importance of long-term MOUD retention and have highlighted how retention can lead to reductions in rates of drug use, hospitalization, criminal activity, and mortality. [23, 29, 30]

Given the rise in methamphetamine and opioid co-use and related morbidity and mortality in North America, including among individuals engaged in MOUD [31], and the importance of MOUD retention for positive health and social outcomes, it is essential to examine the potential impact methamphetamine use may have on MOUD retention. This information can be used to improve treatment responses during the ongoing opioid poisoning crisis. Data on this topic is sparse, with only one identified review which broadly examined the impact of both amphetamines and methamphetamines on receipt of medications for opioid use disorder as well as retention and opioid abstinence, which generally found negative associations between use and retention [32]. Importantly, prior reviews have not synthesized the impact of methamphetamine use on MOUD retention specifically. Therefore, we conducted a scoping review to address this important knowledge gap. Our specific objective was to summarize the available evidence regarding the role of methamphetamine use on MOUD retention among patients enrolled in MOUD.

## Methods

### Definitions

Terms and definitions of opioid pharmacotherapy varied across studies and jurisdictions. Opioid Agonist Treatment (OAT) was the most common and primarily included buprenorphine, buprenorphine/naloxone, and methadone maintenance treatment (MMT). However, some studies used the terms medication-assisted treatment (MAT) and medications for opioid use disorder (MOUD), which includes opioid antagonists (e.g. Naltrexone). Studies also operationalized MOUD retention differently and included dropout/discontinuation from treatment, duration of time spent in treatment, and completion of treatment. For the purposes of this review, we retained the broad term ‘MOUD’ to refer to all opioid pharmacotherapy as the specific medications varied across jurisdictions, and ‘retention’ was defined as any reference to treatment dropout or discontinuation.

### Search strategy

We conducted systematic searches to retrieve studies from scientific literature databases (from database inception to May, 2023): Embase, Medline, PsychINFO, Cochrane Central Register of Controlled Trials (CENTRAL), Cochrane Database of Systematic Reviews, Cochrane Protocols, Cochrane Clinical Answers, CINAHL, Scopus, and Google Scholar (first 200 citations). The search strategy combined MesH terms, Boolean operators, and free-text keywords regarding MOUD and methamphetamine use (see Additional File 1 for an example of the search strategy). In addition, reference lists of included studies were hand-searched, and expert consultations (with SI, JR, FA) were held to identify additional references. No registered review protocol for this scoping review exists.

### Selection criteria

All quantitative studies that examined methamphetamine use regardless of study design, encompassing both experimental and observational designs, were included. Studies were included if all participants in the sample were currently receiving MOUD and the sample included two groups: individuals with methamphetamine use and individuals without methamphetamine use. Using those without methamphetamine use as a control group allowed us to compare MOUD retention between the two groups. Studies in which the sample was comprised of individuals who use methamphetamine on MOUD or those that examined impacts of methamphetamine use among participants unrelated to treatment retention were excluded. No restrictions were applied based on location of studies or date. However, non-peer-reviewed studies, non-empirical studies (commentaries, editorials, opinions, reviews) and non-English language studies were excluded. Studies that reported on ‘amphetamines’ but did not stratify data for methamphetamine specifically were also excluded (See Additional File 2 for flow diagram of study selection).

### Study selection, data extraction, and evidence synthesis

Screening of reports for study selection was carried out in two stages: (1) study title and abstract were screened by two independent screeners (CR and JL) in the first stage, and (2) the full text were reviewed by the same authors for application of selection criteria in the second stage. Interrater reliability for all studies screened was 77.8%, and disagreements and discrepancies were resolved by discussion with a third author (SI). For all included studies, the following data was extracted: study characteristics (authors, publication year, country, study design, data collection time period, and sample size), study sample characteristics (sex, age, and ethnicity), treatment features (type of MOUD and provider details), outcome measures, and main findings. All data were entered into two standardized data extraction charts: one for study characteristics and one for study outcomes. As the outcome definitions of MOUD retention were heterogeneous, findings were presented separately by study in a narrative synthesis.

## Results

### Results of electronic searches

A total of 13,621 articles were retrieved through the database searches. After the removal of duplicates, 10,196 title and abstracts were screened, and 269 full-texts were reviewed for eligibility. A total of eight studies were included in the evidence synthesis.

### Characteristics of included studies

The characteristics of all included studies are detailed in Table 1. Among the eight studies, three took place in Canada (Vancouver, with data collected between 2005–2015, 2005–2018, and 2014–2018, respectively), three took place in the US (Nationally, with data collected in 2017, and two in Washington State, with data collected in 2004–2005 and 2015–2018), while the remaining two were conducted in Iran (Yazd, no data collection time period stated), and China (Guangzhou City, with data collected between 2013–2014).

**Table 1:** Characteristics of Included Studies

Study	Study Setting	Study Design	Time Period	Sex	Age	Ethnicity	Total N	Type of MOUD	Methamphetamine measures
Krawcyk et al. [33]	United States (including District of Columbia and Puerto Rico); all publicly funded/licensed outpatient MOUD treatment facilities	Retrospective analysis	2017	Male: 77,220 (59.26%) Female: 53,062 (40.72%) Missing: 18 (0.01%)	18–29: 39,327 (30.18%) 30–39: 43,038 (33.03%) 40–49: 23,637 (18.14%) 50 + : 24,298 (18.65%)	Non-Hispanic White: 86,031 (66.03%) Non-Hispanic Black: 15,493 (11.89%) Non-Hispanic Other: 6,639 (16.53%) Hispanic (any race): 21,541 (0.46%) Missing: 596 (0.46%) (0.46%)	130,300	(MOUD) including Methadone, Buprenorphine or Naltrexone	Self-reported frequency of methamphetamine use in past month, operationalized as ‘no use’, ‘some use’, ‘daily use’
Liu et al. [38]	Guangzhou, China; four unspecified methadone clinics	Prospective cohort study	2013–2014	Male: 351 (87.5%) Female: 50 (12.5%)	< 40: 117 (29.2%) ≥ 40: 284 (70.8%)	N/A	401	Methadone Maintenance Treatment (MMT)	Self-reported methamphetamine use in the past 6 months, operationalized as ‘yes’ or ‘no’
Lo et al. [37]	Vancouver, Canada; unspecified methadone treatment provider	Prospective cohort study	2005–2015	Male: 792 (60.9%) Female: 508 (39.1%)	Stratified by MMT discontinuation -Median IQ range: Yes: 39 (34–45, 95% CI); No: 42 (35–48, 95% CI)	White: 822 (63.2%) Non-white: 479 (37.2%)	1301	Methadone Maintenance Treatment (MMT)	Self-reported frequency of methamphetamine use in the past 6 months, operationalized as ‘less than daily use’ or ‘more than daily use’
Mackay et al. [34]	Vancouver, Canada; unspecified methadone treatment provider	Prospective cohort study	2014–2018	Male: 495 (57.3%)	Median age (Q1,Q3): 48 (39.5%)	White: 396 (45/6%)	875	Methadone Maintenance Treatment (MMT)	Self-reported frequency of methamphetamine in the past six months, operationalized as ‘more than weekly use’, ‘no more than weekly use’ and ‘not having used any methamphetamine’
Pilarinos et al. [36]	Vancouver, Canada; unspecified methadone treatment provider	Prospective Cohort Study	2005 –2018	Male: 102 (63.8%) Female: 58 (36.3%)	Median age: 24	White: 111(69.4%) Indigenous: 38 (23.8) Other: 11 (6.9%)	160	Methadone Maintenance Treatment (MMT)	Self-reported recent weekly methamphetamine use, operationalized as ‘yes’ or ‘no’
Tsui et al. [35]	Washington State, United States; three publicly funded medication assisted treatment-prescription drug and opioid addiction (WA-MAT-PDOA) clinics	Prospective Cohort Study	2015–2018	Male: 445 (56%) Female: 799 (44.3%)	Mean age: 38.0 (12.2 SD)	White: 638 (80%) Hispanic: 59 (7%) Black: 48 (6%) American Indian: 46 (6%) More than one race: 27 (3%) Asian 13 (2%) Native Hawaiian/Pacific Islander: 12 (2%) Other: 12 (2%) Missing: 3 (0%)	799	Buprenorphine	Self-reported methamphetamine use in past 30 days, operationalized as ‘none’, ‘1–10 days’, ‘11–20 days’, and ‘21–30 days’
Vafeinasab et al. (2015)	Yazd, Iran; unspecified addiction treatment centers	Prospective Cohort Study	N/A	Male: 225 (97.8%) Female: 5 (2.2%)	Mean age: 35 (11–67 years)	N/A	230	Methadone Maintenance Treatment (MMT)	Methamphetamine use verified by rapid diagnostic urine tests
Banta-Green et al. [48]	Washington State, United States; 11 non-profit/public methadone clinics	Retrospective Cohort Study	2004–2005	Female: 1116 (48.4%) Male 1192 (51.7%)	Mean age: 40.6 years (SD 10.3)	Caucasian: 1696 (73.5%)	2308	Methadone Maintenance Treatment (MMT)	Self-reported methamphetamine use in the past 30 days

Seven studies included adults and one included youth (aged 14–26), while seven of the eight studies included both males and females and the one remaining study included only males. Six of the studies included individuals receiving methadone, one included individuals receiving buprenorphine, and one included national data on any MOUD from the national register of treatment discharges in the US. Information pertaining to the study design, age ranges, ethnicity, and study sample is provided in Table 1.

### Evidence synthesis

As the outcome definitions of MOUD retention were heterogeneous, key findings from the included studies are summarized separately below, while analyses, outcome measures, and additional main findings from the studies are detailed in Table 2.

**Table 2:** Analyses, Outcome Measures, and Main Findings of Included Studies

Study	Analysis Type	Outcome Measures	Confounders adjusted for in analyses	Primary outcomes	Primary findings
Krawcyk et al. [33]	1) Multivariate logistic regression and sensitivity analysis 2) Multivariate accelerated time failure model	Retention in MOUD beyond 6 months, based on TEDS length of stay in days	Age Race/ethnicity Education Employment Housing Veteran Status Prior month arrest Age of first use Frequency of use in prior month Primary opioid use Comorbid psychiatric problem Referral source to treatment Other substance use (alcohol, marijuana, benzos, cocaine, methamphetamine)	Comorbid methamphetamine use was associated with lower odds of 6 month treatment retention (Odds Ratio [OR]: 0.48 [95% CI 0.45–0.51]), and shorter 6 month treatment retention (Time Ratio [TR]: 0.64 [95% CI 0.61–0.66]), as well as lower odds of 12 month treatment retention (OR: 0.38 [95% CI 0.35- 0.41]) and shorter 12 month treatment retention (TR: 0.58 [95% CI 0.55–0.60])	Comorbid methamphetamine use was the strongest predictor of shorter MOUD treatment retention, and was strongly associated with lower odds of 6 month and 12 month MOUD retention
Liu et al. [38]	1) Cox regression models to predict drop-out 2) Log-binomial regression models to predict poor adherence	Drop-out of MMT: not having visited the clinic for at least 30 consecutive days prior to the study’s completion date Poor-adherence to MMT: either drop-out case or having attended MMT clinic for less than 50% of the follow-up period to cover intermittent MMT	Gender Age Education level Marital status Employment status HIV infection status Drug use history MMT history	Those who used methamphetamine in the past 6 months had a higher likelihood of MMT treatment dropout( Adjusted Hazard Ratio [aHR]: 2.26 [95% CI 1.15–4.43])	Patients who had used methamphetamine or any kind of club drugs in the last 6 months were 2.26 times more likely than others to drop out from MMT
Lo et al. [37]	1) Pearson’s Chi-Square for categorical variables 2) Mann–Whitney rank sum for continuous variables 3) GEE analyses	Self-reported MMT discontinuation in the last 6 months, defined as accessing methadone at one visit, and not being on methadone at a subsequent visit	Age Gender Race Homelessness status HIV status Substance use history (alcohol, crack cocaine, opioids, heroin, cocaine) MMT history	Those who used methamphetamine daily were more likely to discontinue MMT treatment (OR:1.75 [95% CI 1.07–2.85])	Daily methamphetamine use was associated with 1.75 times likelihood of MMT discontinuation
Mackay et al. [34]	1) Poisson distribution 2) Chi-squared and Wilcoxon Rank Sum 3) Kaplan–Meier to determine probability of methadone discontinuation (4) Bivariable and multivariable Cox regression models	Time to discontinuation of methadone, defined as not being on methadone at the time of a follow-up interview during study period	Age Gender Self-identified ancestry HIV serostatus Incarceration history Living in the downtown east side(DTES) Homelessness history Other substance use (opioid, cocaine, crack, alcohol) Methadone history	Those who reported more than weekly methamphetamine use were more likely to discontinue treatment **(**aHR: 1.38 [95% CI 1.03–1.85]) In a sub-analysis, compared to no methamphetamine use, all routes of administration of methamphetamine were significantly associated with methadone discontinuation: both injection and non-injection (HR: 1.97 [95% CI 1.40–2.77]), non-injection only (HR: 1.85 [95% CI 1.20–2.86), and injection only (HR: 1.75 [95% CI 1.29–2.38])	Compared to no methamphetamine use, at least weekly methamphetamine use was independently associated with higher rates of methadone discontinuation. All routes of administration of methamphetamine were significantly associated with methadone discontinuation
Pilarinos et al. [36]	(1) Chi-squared (2) Mann–Whitney (3) Bivariate and multivariable Cox regression models	Time to any MMT discontinuation, defined as individuals who indicated they had received MMT in the last 6 months but were not currently on MMT	Age Age of first drug use Sex Race (Indigeneity) Ethnicity Depression Child welfare involvement Childhood adverse events MMT initiation period Recent living in downtown east side (DTES) Recent drug use Recent employment Recent homelessness Recent incarceration Recent non-pharmacological treatment Recent difficulty accessing services	Those who reported recent weekly crystal methamphetamine use were more likely to discontinue treatment (aHR: 1.67 [95% CI 1.19–2.35]) In sub-analyses, recent weekly crystal methamphetamine use was also positively associated with ‘actionable’ MMT discontinuation in adjusted analyses (aHR = 4.61 [95% CI 1.78–11.9])	Self-reported weekly use of crystal methamphetamine is associated with an increased likelihood of MMT treatment dropout, as well as ‘actionable’ dropout (i.e., reason for dropout that can be addressed through policy or guideline changes)
Tsui et al. [35]	(1) Kaplan–Meier survival curves to assess association between methamphetamine use and time to discharge (2) Cox proportional hazards regression used to estimate the relative hazards for treatment discharge	Survival time defined as time from buprenorphine induction/enrollment to earliest date of discharge Primary outcome: time to buprenorphine treatment discharge (if no active prescription for buprenorphine and no contact with program for > 30 days)	Age Gender Clinic site Period of enrollment in treatment Race Ethnicity Education level Non-methamphetamine substance use Previous treatment history	Those with past-month methamphetamine use at baseline were more likely to drop out of buprenorphine treatment (HR: 2.39 [95% CI 1.94–2.93]); the risk increased with additional days of methamphetamine use: 1–10 days (HR:2.05 [95% CI1.63–2.57]); 11–20 days (HR: 3.04 [95% CI2.12–4.23]); 21–30 days (HR: 3.61 [95% CI 2.40–5.23])	Methamphetamine use is associated with increased risk of non-retention for patients who are treated for OUD with buprenorphine. The risk increases with additional days of methamphetamine use
Vafaeinasab et al. [39]	(1) Chi-square test and survival analysis (2) Log-rank and Kaplan–Meier curves	Methadone consumption and therapy/discontinuation of treatment recorded by physician or consultant; Therapy survival rates calculated at first, third, and 6 months	Gender Relationship with family/family support Age Treatment history Substance use history Physical and mental illness history	A lower proportion of individuals who had positive urinalysis for methamphetamine were retained in methadone at 6 months, however, findings were not significant due to low sample size. A total of 14.8% of individuals who had at least one positive test of methamphetamine use continued treatment for up to 6 months, compared to 30.2% of individuals who did not test positive for methamphetamines	Patients with at least one positive test for methamphetamine use during treatment period experienced lower rates of retention in treatment
Banta-Green et al. [48]	(1) Bivariate statistics (chi-squared and t-tests) (2) Logistic regression model	12 month treatment retention, defined as remaining in treatment at day 366 following admission to MMT	Age Marital status Educational status Race Public assistance type Medical severity/psychiatric severity composite score Housing status Current legal involvement Drug use at time of intake	Those who reported methamphetamine were less likely to be retained in MMT treatment at 12 months (OR:0.62 [95% CI 0.44–0.89])	Methamphetamine use was significantly associated with decreased odds of MMT retention

Krawczyk et al. [33] examined retention in MOUD treatment beyond six months drawing on a national US sample of publicly licensed/funded substance use treatment facilities, based on the variable ‘length of stay in treatment (days)’ which was computed using the date of admission and the date of last contact from a US database of treatment admissions. Treatment discharges were recorded as the date of last contact, and reasons for discharge were provided and comprised ‘treatment completed’, ‘dropped out of treatment’, ‘terminated by facility’, ‘transferred to another program/facility’, ‘incarcerated’, or ‘other reason’. Risk factors and drivers of treatment retention were explored. The authors adjusted their analyses to account for sociodemographic variables as well as treatment use and substance use history. Comorbid methamphetamine use was associated with lower odds of both 6 month treatment retention (Odds Ratio [OR]: 0.48 [95% Confidence Interval [CI] 0.45–0.51]) and 12 month treatment retention (OR: 0.38 [95% CI 0.35–0.41]), as well as shorter duration of treatment overall.

Mackay et al. [34] examined self-reported past 6 month methamphetamine use on time to methadone discontinuation among individuals engaged in methadone in Vancouver, Canada. Methadone discontinuation was defined as being on methadone at the first study visit and subsequently not being on methadone at follow-up. In unadjusted bivariate analyses, both ‘no more than weekly use’ (Hazard Ratio [HR]: 1.49, [95% CI 1.08–2.04]), and ‘more than weekly use’ (HR: 2.17 [95% CI 1.63–2.88]) were significantly associated with methadone discontinuation. In adjusted multivariate analyses, which included controlling for secondary sociodemographic and substance use variables, as well as prior treatment history, ‘weekly or more’ methamphetamine use remained associated with methadone discontinuation (Adjusted Hazard Ratio [aHR]: 1.38 [95% CI 1.03–1.85]). Moreover, compared to no methamphetamine use, all routes of administration of methamphetamine were significantly associated with methadone discontinuation: both injection and non-injection (HR: 1.97 [95% CI 1.40–2.77]), non-injection only (HR: 1.85 [95% CI 1.20–2.86]), and injection only (HR: 1.75 [95% CI 1.29–2.38]) (non-injection primarily includes inhalation).

Tsui et al. [35] examined the impact of self-reported past-month methamphetamine use on buprenorphine treatment retention, measured at baseline and then again at 6 months, among a sample of individuals receiving buprenorphine in Washington, US. The main outcome was treatment discharge, defined as not having an active buprenorphine prescription or contact with the program for more than 30 days. The authors adjusted for clinic site, time period of enrollment, and sociodemographic characteristics such as age, gender, ethnicity, and race. Past 30 day baseline methamphetamine use was associated with a two times relative hazard ratio (aHR: 2.39 [95% CI 1.94–2.93]) to be discharged from treatment at 6 month follow-up. Moreover, the magnitude of the effect size increased with frequency of use, with those using on 1–10 days of the month experiencing over two times the risk of discharge (HR: 2.05 [95% CI 1.63–2.57]), those using 11–20 days experiencing over three times the risk of discharge (HR: 3.04 [95% CI 2.12–4.23]), and those using 21–30 days experiencing more than 3.5 times the risk of treatment discharge (HR: 3.61 [95% CI 2.40–5.23]). [35]

Pilarinos et al. [36] examined factors associated with time to methadone discontinuation among a sample of youth (aged 14–26) engaged in methadone in Vancouver, Canada. Discontinuation was defined as individuals who indicated they had received MMT in the last 6 months but were not currently on MMT during data collection. In adjusted analyses, the authors found that MMT discontinuation was positively associated with self-reported weekly crystal methamphetamine use among youth (aHR: 1.67 [95% CI 1.19–2.35,]). In adjusted sub-analyses, recent weekly crystal methamphetamine use was also positively associated with ‘actionable’ MMT discontinuation (aHR = 4.61 [95% CI 1.78–11.9]), meaning the reason for discontinuation could be addressed through policy or guideline changes.

Lo et al. [37] examined self-reported methadone continuers (defined as individuals currently on methadone at the time of study assessment) versus discontinuers (defined as individuals who were on methadone during the last study assessment but were not currently on methadone during data collection) among individuals receiving methadone in Vancouver, Canada. The main outcome was treatment discontinuation in the past 6 months. In unadjusted bivariate analyses, clients who used methamphetamine daily were 1.75 times more likely (OR: 1.75 [95% CI 1.07–2.85]) to become discontinuers of methadone; however, these findings were non-significant when adjusted for sociodemographic information, housing conditions, criminal justice history, and HIV status (aOR: 1.02 [95% CI 0.61–1.69]).

Liu et al. [38] examined self-reported methamphetamine use during the past 6 months in relation to methadone treatment dropout and poor treatment adherence among a sample of individuals engaged in methadone treatment in Guangzhou, China. Treatment dropout was defined as not visiting the treatment facility consecutively for 30 days prior to treatment completion. After adjusting for confounding variables such as sociodemographic characteristics, drug use history and prior treatment history, patients who used methamphetamine were 2.6 times more likely to drop out of treatment than patients who did not use methamphetamine (aHR: 2.26 [95% CI 1.15–4.43]). Poor treatment adherence was defined as attending treatment facility for less than 50% of the follow-up period.

Vafaeinasab et al. [39] examined methadone treatment survival observed at 1 month, 2 months, and 6 months of treatment among individuals receiving methadone in Yazd, Iran. The main outcome was treatment discontinuation, which was defined as ‘absence of therapy or discontinuation of treatment’. Methamphetamine use was measured using urinalysis. Sociodemographic characteristics, family and judicial status, physical and mental illness, drug use history, and treatment history were also recorded. A lower proportion of individuals who had positive urinalysis for methamphetamine were retained in methadone at 6 months, however, findings were not significant due to low sample size. A total of 14.8% of individuals who had at least one positive test of methamphetamine use continued treatment for up to 6 months, compared to 30.2% of individuals who did not test positive for methamphetamines.

Lastly, Banta-Green et al. [40] examined the impact of self-reported methamphetamine use at intake on methadone retention among individuals in methadone treatment in Washington, US. The main outcome was 12 month methadone retention, which was defined as remaining in treatment at day 366 following admission to methadone. After adjusting for covariates including sociodemographic characteristics, psychiatric composite severity score, and substance use at time of intake, those who reported methamphetamine use at intake were significantly less likely than those that did not to remain in methadone at 12 months (OR: 0.62 [95% CI 0.44–0.89]).

## Discussion

### Summary of findings

This scoping review examined the impact of methamphetamine use on MOUD retention. All eight studies identified a higher likelihood of treatment discontinuation or dropout among individuals in MOUD who used methamphetamine, underscoring the strong and significant potential negative impact of methamphetamine use on treatment retention. Furthermore, two studies found that frequency of methamphetamine use was a significantly associated with MOUD dropout; those who use methamphetamine more frequently are less likely to remain in treatment compared to those who use less frequently. Additionally, one study found that different routes of methamphetamine administration -non-injection (inhalation), injection, or both -were all significantly associated with treatment dropout, with those who inject *and* inhale methamphetamine being slightly more likely to drop out of treatment than those who just inject or just inhale methamphetamine. [34]

### Comparison with prior research

Although very few studies have examined the impact of methamphetamine use on MOUD outcomes, our study results corroborate extant literature highlighting the negative impact of substance–and particularly stimulant–use on MOUD outcomes [32]. For instance, individuals with a positive cocaine urinalysis at baseline were more likely to leave buprenorphine treatment earlier and discontinue treatment within 6 months [41]. In a multisite study comparing treatment retention between those engaged in buprenorphine/naloxone versus methadone, use of amphetamines or cocaine was associated with treatment dropout and shorter retention among both groups [42]. In an another study, both baseline and continued cocaine use among individuals engaged in MOUD was predictive of treatment dropout, and frequency of use was positively associated with decreases in treatment retention. [43]

Beyond MOUD retention, available evidence suggests high and increasing levels of concurrent methamphetamine and opioid use are occurring among those engaged in or entering MOUD. For example, Dong et al. (2020) found high proportions of concurrent stimulant use (including cocaine, crack cocaine and crystal methamphetamine) among individuals entering MOUD treatment, with 74–91% of individuals who use stimulants reporting using opioids [44]. Cui et al. (2022) similarly observed a rapidly increasing pattern of crystal methamphetamine use between 2005 and 2020, and a higher crystal methamphetamine use frequency particularly among individuals who reported ongoing unregulated opioid use and who initiated MOUD [45]. In a separate study, Cui et al. (2022) also identified a 7% yearly increase in the rate of crystal methamphetamine initiation or re-initiation among individuals on MOUD in Vancouver, Canada, suggesting a three-fold increase compared to a decade ago (23.2/100,000 person years in 2019 vs. 7.6/100,000 person years in 2008) [46]. It has been postulated that some individuals engaged in MOUD may make compensatory adjustments such as substituting with methamphetamine to counteract the lack of pleasure typically experienced with illicit opioid use, or due to the reduction in illicit opioid use following MOUD engagement, particularly if they have not achieved a stable or comfortable MOUD dose [20]. As an example of this potential, one study that examined 30 year substance use trajectories among individuals engaged in methadone treatment found that those who quickly reduced their opioid use post-MOUD engagement subsequently increased their concurrent use of amphetemines [47]. The increase in methamphetamine use could ultimately result in increased risk for MOUD dropout, highlighting the potential cyclical nature of methamphetamine use on MOUD engagement.

While smoking may have traditionally been the dominant route of administration for methamphetamine use, available data indicate that injection methamphetamine use is on the rise [9]. For instance, among individuals presenting to treatment in the US for methamphetamines, injection as the main route of administration increased from 18% to 28.2% of all admissions between 2010 and 2019 [9]. Additional cross-sectional data from Washington revealed that the proportion of individuals reporting methamphetamine injection increased from 20 to 65% between 2009 and 2017 among needle exchange program clients, with most of the increase attributable to the co-injection of heroin/opiates and methamphetamines (commonly referred to as a ‘goofball’) [48–52]. Goofball use may pose additional risks and has been associated with higher substance use risk profiles including sharing substance use equipment [48, 53]. Moreover, compared to those who only inject heroin, goofball use has been associated with a nearly threefold increase in past-year prevalence of poisonings [49], and past 6-month poisonings have been significantly associated with higher odds of heroin/methamphetamine co-injection [53]. Risks related to the route of administration of methamphetamine is thus an important factor to consider in regard to MOUD retention, particularly given the high risks associated with injection and the significant association that was found between all routes of methamphetamine administration and MOUD dropout in the one study that examined this. [34]

### Implications

The results of our study demonstrate that methamphetamine use during MOUD can lead to negative treatment outcomes including reduced retention in MOUD, which has substantial clinical implications. In the backdrop of the ongoing and evolving opioid poisoning crisis, there is an increased need to identify methamphetamine use among those entering MOUD as a particular risk factor for treatment drop out. Current evidence-based MOUD treatment guidelines in both Canada and the US suggest that use of other drugs (including stimulants in particular) during MOUD treatment is not an appropriate reason for withholding or discontinuing treatment [54, 55]. Individuals engaging in co-use should still be encouraged to initiate MOUD and be provided with education on the risks of methamphetamine use during treatment, including the increased risk of treatment dropout. In addition, services and programs for individuals who concurrently use methamphetamine and opioids should be designed, implemented, and evaluated. Compounding the problem at hand is that evidence-based treatments (including pharmacotherapy options) for methamphetamine are limited, unlike opioids [56, 57]. For instance, there are no approved pharmacotherapies for methamphetamine use disorder, and available evidence indicates that most medications have shown no statistically significant benefit [57], and treatment options are inadequate [58]. Some treatment options studied previously that have shown partial positive results include psychosocial and pharmacological treatments such as contingency management, cognitive behavioral therapy, dopamine agonists, antipsychotics, and opioid agonists [58]. Most recently, the combination of extended-release injectable naltrexone plus oral extended-release bupropion has shown promise [59]. Based on available evidence, MOUD practitioners should refer patients who use methamphetamines while on MOUD to health care providers who can provide adjunct psychosocial and behavioral treatment in order to reduce the potential negative impact of methamphetamine use on MOUD retention, and improve overall outcomes.

## Limitations

The findings should be considered in the context of some limitations. Despite the broad search strategies in multiple electronic databases, a small number of studies were available on the topic. For the purposes of this paper, the vast heterogeneity of included studies in terms of the outcomes, differences in pharmacotherapies (e.g., methadone versus buprenorphine versus MOUD), as well as treatment regimen, limited the applicability of conducting a meta-analysis. There was also variance in methamphetamine measures used, and no studies examined co-occurring opioid and methamphetamine use disorder. As such, the results were narratively summarized. Furthermore, generalizability beyond the contexts of studies within which they took place is limited. Specifically, MOUD formulations and programming vary drastically from jurisdiction to jurisdiction, with strict prescribing practices in the US compared to Canada, and limited formulations and options in the other global regions (e.g. China and Iran), including an overreliance on criminal justice referrals to treatment. Future studies should examine the context of treatment being studied, different opioid treatment formulations, additional outcomes (e.g. poisoning, mortality, hospitalizations, and criminal justice outcomes), and sociodemographic differences in impacts. Additionally, studies should consider conducting a meta-analysis to pool results. Most studies were conducted prior to the increase in polysubstance and methamphetamine use that has recently occurred in the North American context. The latest data included in the studies was collected in 2018. As such, these studies are likely not reflective of current substance use trends, and may potentially underestimate the impact methamphetamine use may have on present-day MOUD retention.

## Conclusion

In the context of the rise in methamphetamine and opioid co-use and related harms in North America, it is important to understand the potential impact of methamphetamine use on MOUD outcomes, including treatment retention. Our scoping review found that methamphetamine use reduces MOUD retention, with evidence of a dose-dependent effect of increasing likelihood with increased frequency of use. Strategies to identify concurrent methamphetamine use and educate individuals on the increased risk of treatment drop out should be undertaken.

### Supplementary Information

**Additional file 1: **Initial search strategy.

**Additional file 2: **PRISMA chart.

## Data Availability

All data generated or analysed during this study are included in this published article [and its Additional file information files].

## Abbreviations

OAT: Opioid agonist treatment
OUD: Opioid use disorder
US: United States
BC: British Columbia
HIV: Human immunodeficiency virus
HCV: Hepatitis C Virus
MMT: Methadone maintenance treatment
MAT: Medication-assisted treatment
MOUD: Medications for opioid use disorder
