# Supplementary material for: The impact of methamphetamine use on medications for opioid use disorder (MOUD) treatment retention: a scoping review
Source: Addict Sci Clin Pract. 2023 Aug 16;18:48. doi: 10.1186/s13722-023-00402-0 (PMC10433668; doi:10.1186/s13722-023-00402-0)
Supplement: Supplementary file 1 — Additional file 1: Initial search strategy. [file 13722_2023_402_MOESM1_ESM.docx]

# Additional File 1: Initial Search Strategy

# Embase Classic+Embase

Embase Classic+Embase <1947 to 2023 May 26>

**5676 total**

1 exp opiate antagonist/ or exp opiate addiction/ or opiate.mp. or exp opiate agonist/ or exp opiate/ or exp opiate substitution treatment/ 579209

2 opioid*.mp. 165904

3 exp buprenorphine/ or exp methadone/ or exp opiate addiction/ or opioid addict*.mp. or exp diamorphine/ 93424

4 "opioid use disorder".mp. 8749

5 opioid agonist treatment.mp. 775

6 suboxone.mp. or exp buprenorphine plus naloxone/ 2762

7 sublocade.mp. or exp buprenorphine/ 25221

8 exp methadone treatment/ or exp maintenance therapy/ or exp methadone/ or methadone.mp. or exp methadone plus naloxone/ 109442

9 exp opiate substitution treatment/ or opioid substitution.mp. 3715

10 opioid pharmacotherapy.mp. 128

11 buprenorphine maintenance.mp. 472

12 exp naltrexone/ or injectable opioid agonist treatment.mp. or exp morphine/ or exp hydromorphone/ 142802

13 vivitrol.mp. or exp naltrexone/ 16990

14 subutex.mp. or exp buprenorphine/ 25233

15 extended-release.mp. 12695

16 medication assisted treatment.mp. 1379

17 "MAT".mp. 15913

18 1 or 2 or 3 or 4 or 5 or 6 or 7 or 8 or 9 or 10 or 11 or 12 or 13 or 14 or 15 or 16 or 17 693292

19 exp methamphetamine dependence/ or exp methamphetamine/ or methamphetamine.mp. 27065

20 "Meth".mp. 7104

21 "crystal methamphetamine".mp. 408

22 "crystal meth".mp. 222

23 methylamphetamine.mp. 1350

24 "methamphetamine use".mp. 2507

25 19 or 20 or 21 or 22 or 23 or 24 31495

26 18 and 25 7754

27 limit 26 to (human and english language)

# MEDLINE

Ovid MEDLINE: Epub Ahead of Print, In-Process & Other Non-Indexed Citations, Ovid MEDLINE® Daily and Ovid MEDLINE® <1946-2023 May 26>

**1653 total**

1 opiate*.mp. or exp Opiate Substitution Treatment/ 31257

2 exp Opioid-Related Disorders/ or opiate agonist.mp. 34437

3 exp Naltrexone/ or opiate antagonist.mp. 9995

4 opiate addiction.mp. 905

5 opioid*.mp. or exp Analgesics, Opioid/ 202320

6 "opioid use disorder".mp. 6449

7 exp Methadone/ or opioid agonist treatment.mp. or exp Buprenorphine/ 19524

8 suboxone.mp. or exp Buprenorphine, Naloxone Drug Combination/ 665

9 exp Buprenorphine/ or sublocade.mp. 7321

10 methadone.mp. or exp Methadone/ 18783

11 methadone treatment.mp. 1752

12 maintenance therapy.mp. 15639

13 opioid substitution.mp. 1029

14 opioid pharmacotherapy.mp. 97

15 buprenorphine maintenance.mp. or exp Heroin Dependence/ 9608

16 exp Heroin/ or injectable opioid agonist treatment.mp. or exp Hydromorphone/ 7732

17 exp Morphine Dependence/ or morphine.mp. or exp Morphine/ 64339

18 exp Narcotic Antagonists/ or exp Naltrexone/ or vivitrol.mp. 41406

19 subutex.mp. or exp Buprenorphine/ 7328

20 exp Delayed-Action Preparations/ or extended-release.mp. 55202

21 medication assisted treatment.mp. 960

22 "MAT".mp. 12037

23 1 or 2 or 3 or 4 or 5 or 6 or 7 or 8 or 9 or 10 or 11 or 12 or 13 or 14 or 15 or 16 or 17 or 18 or 19 or 20 or 21 or 22 309081

24 methamphetamine.mp. or exp Methamphetamine/ 16068

25 exp Amphetamine-Related Disorders/ or methamphetamine dependence.mp. 3669

26 "meth".mp. 8826

27 crystal methamphetamine.mp. 306

28 "crystal meth".mp. 140

29 methylamphetamine.mp. 750

30 "methamphetamine use".mp. 1900

31 24 or 25 or 26 or 27 or 28 or 29 or 30 23954

32 23 and 31 2516

33 limit 32 to (english language and humans) 1653

**ProQuest – PsychINFO**

APA PsycInfo <1806 to May Week 4 2023>

**2608 Total**

1 exp Methadone Maintenance/ or exp Heroin/ or exp Morphine/ or exp Drug Dependency/ or opiate addiction.mp. or exp Heroin Addiction/ or exp "Substance Use Treatment"/ or exp Drug Addiction/ or exp Opiates/ or exp Drug Abuse/ or exp Narcotic Agonists/ 119144

2 exp Analgesic Drugs/ or exp "Opioid Use Disorder"/ or opioid*.mp. 53042

3 exp Buprenorphine/ or exp Methadone Maintenance/ or exp Methadone/ or opioid agonist treatment.mp. 7525

4 exp Buprenorphine/ or suboxone.mp. or exp Pharmacology/ 28968

5 exp Buprenorphine/ or exp Naloxone/ or buprenorphine plus naloxone.mp. 5168

6 methadone.mp. 8928

7 opioid substitution treatment.mp. 285

8 maintenance therapy.mp. or exp Maintenance Therapy/ 6045

9 opioid pharmacotherapy.mp. 49

10 opioid antagonists.mp. or exp Narcotic Antagonists/ 6612

11 naltrexone.mp. or exp Naltrexone/ 4653

12 buprenorphine maintenance.mp. 274

13 injectable opioid agonist treatment.mp. 17

14 exp Morphine Dependence/ or exp Morphine/ or morphine.mp. 12040

15 exp Medication-Assisted Treatment/ or vivitrol.mp. 4181

16 exp Buprenorphine/ or subutex.mp. 2357

17 extended-release.mp. 1810

18 "MAT".mp. 1323

19 1 or 2 or 3 or 4 or 5 or 6 or 7 or 8 or 9 or 10 or 11 or 12 or 13 or 14 or 15 or 16 or 17 or 18 165193

20 methamphetamine.mp. or exp Methamphetamine/ 7052

21 methamphetamine dependence.mp. 446

22 crystal methamphetamine.mp. 270

23 crystal meth.mp. 104

24 methylamphetamine.mp. 266

25 "methamphetamine use".mp. 1406

26 meth.mp. 1374

27 20 or 21 or 22 or 23 or 24 or 25 or 26 7409

28 19 and 27 3286

29 limit 28 to (english and human) 2608
