# Supplementary material for: The impact of methamphetamine use on medications for opioid use disorder (MOUD) treatment retention: a scoping review
Source: Addict Sci Clin Pract. 2023 Aug 16;18:48. doi: 10.1186/s13722-023-00402-0 (PMC10433668; doi:10.1186/s13722-023-00402-0)
Supplement: Supplementary file 2 — Additional file 2: PRISMA chart. [file 13722_2023_402_MOESM2_ESM.docx]

# Additional File 2: PRISMA Chart

Studies included in synthesis (meta-analysis)
(n=8)

## Included

## Eligibility

## Screening

## Identification

Records excluded
(n=8,943)

Records screened
(n=10,196)

Records after duplicates removed
(n=10,196)

Additional records identified through other sources
(n = 1)

Records identified through database searching
(n=13,621)

Full-text articles assessed for eligibility
(n=269)

Full-text articles excluded, with reasons
(n=261)

*From:*  Moher D, Liberati A, Tetzlaff J, Altman DG, The PRISMA Group (2009). *P*referred *R*eporting *I*tems for *S*ystematic Reviews and *M*eta-*A*nalyses: The PRISMA Statement. PLoS Med 6(7): e1000097. doi:10.1371/journal.pmed1000097

**For more information, visit** [**www.prisma-statement.org**](http://www.consort-statement.org/)**.**
